# Molecular Detection and VP2 Characterization of Feline and Canine Parvoviruses in Domestic Cats in Central Vietnam

**DOI:** 10.3390/ijms27198541

**Published:** 2026-09-24

**Authors:** Giang Huong Thi Tran, Ngan Hong Thi Le, Amonpun Rattanasrisomporn, Jatuporn Rattanasrisomporn

**Affiliations:** 1 Faculty of Veterinary Medicine, Vietnam National University of Agriculture, Hanoi 131000, Vietnam; tthgiang@vnua.edu.vn; 2 Dak Lak Sub-Department of Livestock Production and Animal Health, Buon Ma Thuot Ward 63000, Dak Lak, Vietnam; ngancnty11@gmail.com; 3 Interdisciplinary of Genetic Engineering and Bioinformatics, Graduate School, Kasetsart University, Bangkok 10900, Thailand; fgraapr@ku.ac.th; 4 Department of Companion Animal Clinical Sciences, Faculty of Veterinary Medicine, Kasetsart University, Bangkok 10900, Thailand

**Keywords:** feline panleukopenia virus (FPV), canine parvovirus (CPV), genetic characterization, PCR, Vietnam

## Abstract

In this study, 269 fecal specimens were sampled from domestic cats displaying symptomatic and asymptomatic individuals in Daklak and Gialai provinces in Central Vietnam during July 2023 to July 2025. The polymerase chain reaction method was applied to determine the feline parvovirus DNA in the obtained samples. Positive samples were randomly chosen for full-length VP2 gene sequencing using the Sanger method. The feline parvovirus DNA was detected in 25 (9.29%) of 269 samples. The viral DNA-positive rate was 17.65% (15/85) from cats aged below 6 months, significantly higher than that from 6–12- and >12-month-old cats. Seven complete VP2 sequences were successfully obtained from the identified strains, exhibiting a high degree of nucleotide identity (98.23–100%). Comparison to that of other sequences retrieved from GenBank indicated that seven current Vietnamese sequences displayed the highest nucleotide identity was from 99.08 to 99.94 with that of viral strains from China, Italy, and Spain. Phylogenetic analysis constructed using the complete VP2 gene sequences indicated that six Vietnamese sequences obtained belonged to feline panleukopenia virus (FPV) lineage and divided into several clusters, which were closely related to viruses from China, Italy, and Spain. The remaining one sequence was defined as canine parvovirus (CPV) which was genetically related to previous Vietnamese CPV-2c and Chinese strains. Twenty-six negative selection sites were detected in the VP2 protein of the current Vietnamese FPV/CPV strains in this study.

## 1. Introduction

Feline parvovirus (FPV; synonymous with feline panleukopenia virus or feline viral enteritis) is a highly transmissible viral pathogen of major clinical importance in domestic cats, frequently causing severe disease and death in advanced cases [1,2]. The virus can infect all types of cats, both felid species and certain non-felid hosts such as raccoons and minks [1]. Among affected populations, kittens are particularly susceptible, as their immature immune systems are less capable of mounting an effective defense, resulting in higher mortality rates [3,4].

Clinical signs of the disease range widely, from mild and self-limiting illness to severe conditions and rapidly fatal outcomes [2]. Infected cats typically display symptoms such as lethargy, anorexia, vomiting, diarrhea, and marked dehydration [5]. Clinically, the disease is divided into four forms, including peracute, acute, subacute, and perinatal. Additionally, both morbidity and mortality rates are influenced by the virulence of the infectious strain [6]. FPV exhibits a high level of environmental stability and resistance, demonstrating resistance to extremely low temperatures and many commonly used disinfectants, which enables its prolonged persistence in contaminated environments [7].

FPV, classified within the *Parvoviridae* family, is a non-enveloped, single-stranded DNA virus of small size. It shows high similarity with canine parvovirus (CPV) in terms of its genome, structure, and immunological characteristics. CPV is divided into several types, 2a, 2b, and 2c, which can also infect cats. It is widely hypothesized that FPV is the original virus from which CPV evolved through natural mutations [8]. In the *Parvovirus* genus, FPV, CPV, and mink enteritis virus (MEV) constitute a closely related antigenic cluster [8,9,10,11].

With a genome size of about 5.2 kb in size, FPV contains four encoded open reading frames (ORFs): two capsid proteins (VP1 and VP2) and two non-structural proteins (NS1 and NS2) [12,13]. Among them, the VP2 protein, encoded by the fourth ORF, represents about 90% of the viral capsid and plays a critical role in defining host specificity. It also contains multiple B-cell epitopes that are essential for eliciting neutralizing, protective antibody responses [14]. Due to its importance in viral evolution, host adaptation, and immune recognition, the VP2 gene has been extensively investigated and is commonly used as a target antigen in the development of subunit vaccines [14,15].

FPV and CPV have been widely reported in companion animals worldwide. In Vietnam, parvovirus has been reported [16,17,18]; however, the available data on FPV and CPV are still limited, particularly in Central Vietnam, where their genetic characteristics and evolutionary relationships have not been well characterized. Therefore, this study provides a systematic analysis of the molecular features of FPV and CPV in cats from Central Vietnam based on VP2 sequences, which is necessary to address an important gap in the molecular epidemiology of FPV and CPV in the region.

## 2. Results

### 2.1. Identification of the Parvovirus Genome by PCR

This study involved the collection of samples from domestic cats in Central Vietnam. Of 269 tested samples, 25 (9.29%) samples were PCR-positive for the viral DNA. Specifically, the parvovirus DNA-positive rates were 5.16% and 25% in the Daklak and Gialai provinces, respectively (Table 1), which indicated a significant difference in the feline parvovirus DNA detection rates between the two provinces (*p* < 0.001). Interestingly, vaccination status was unavailable for any of the cats in the current study, including the 25 positive samples.

Additionally, the distribution of the *Parvovirus* genome in the collected samples was further analyzed with respect to breed, age, sex, and health status (Table 2). In detail, the parvovirus genome was found in all age categories, including <6 months, 6–12 months, and >12 months. Notably, the occurrence rate in cats younger than 6 months (17.65%) was significantly higher than that in cats aged 6 to 12 months (4.24%) (*p* = 0.003). Cats aged >12 months had a genome-positive rate of 7.58%, which was lower than that observed in cats aged <6 months and higher than that in cats aged 6–12 months, although the differences were not statistically significant (*p* = 0.09 and *p* = 0.33, respectively). In contrast, no significant differences were observed across breed, sex, and health status. Briefly, the viral genome was detected in 23/216 native cats (10.65%) and 2/53 exotic or cross-bred cats (3.77%). Furthermore, parvovirus was also identified in both male (7.96%) and female (10.26%) as well as in healthy (8.02%) and sick cats (14.04%).

### 2.2. Analysis of the Complete VP2 Gene of Vietnamese Parvovirus Strains

To investigate the genetic characteristics of the viral strains, seven *Parvovirus* DNA-positive samples obtained from domestic cats in two provinces, Daklak and Gialai, were subjected to nucleotide sequencing. The sequencing chromatograms exhibited distinct peaks corresponding to individual nucleotides, indicating the high quality and reliability of the sequencing data. The complete VP2 gene sequences were generated by assembling three overlapping amplicons obtained using separate primer pairs and independently verified by duplicate sequencing, ensuring full-length coverage of the VP2 gene. In the current study, among the 25 PCR-positive samples, seven samples were selected for complete VP2 gene sequencing based on the quality and quantity of PCR products sufficient for sequencing and to provide representation from the two neighboring provinces. The selected samples represented three samples from Gialai and four samples from Daklak. The obtained VP2 gene sequences of the Vietnamese strains were named Cat/Vietnam/FPV-334/2024, Cat/Vietnam/FPV-340/2024, Cat/Vietnam/FPV-343/2024 Cat/Vietnam/FPV-351/2025, Cat/Vietnam/FPV-361/2025, Cat/Vietnam/CPV-455/2025, and Cat/Vietnam/FPV-465/2025. For brevity, the strain names were abbreviated as FPV-334/2024 (PZ276925), FPV-340/2024 (PZ276926), FPV-343/2024 (PZ276927), FPV-351/2025 (PZ276928), FPV-361/2025 (PZ276929), CPV-455/2025 (PZ276931), and FPV-465/2025 (PZ276930), respectively. Sequence analysis revealed that six parvovirus strains were determined as FPV, whereas one parvovirus strain was clustered within CPV.

Seven Vietnamese parvovirus strains with complete VP2 gene sequences (1755 bp) were aligned and analyzed alongside reference sequences available in GenBank. The nucleotide sequence similarity among these strains ranged from 98.23% to 100% (Table 3). Notably, strains FPV-334/2024, FPV-340/2024 and FPV-361/2025 were identical at the nucleotide level (100%), whereas strains FPV-465/2025 and CPV-455/2025 exhibited the lowest level of similarity (98.23%) (Table 3A). At the protein level, identities of VP2 amino acid (aa) sequences ranged from 98.22% to 100% (Table 3B).

Comparative evaluation of the complete VP2 gene sequences from the seven obtained parvovirus strains and GenBank reference sequences revealed high nucleotide similarity. Strains FPV-343/2024 and FPV-351/2025 exhibited 99.77% identity with the JSYZ-122 strain (Accession no. MW017628.1) reported in China in 2020 (Table 4). Strains FPV-334/2024, FPV-340/2024, and FPV-361/2025 shared the highest nucleotide similarity (99.94%) with the YZ2019-025 strain (Accession no. OQ863617.1), originally reported in China in 2019. Among the current Vietnamese strains, the FPV-465 strain displayed 99.85% nucleotide identity with the Felocell strain (Accession no. EU498681.1) reported in Italy in 2008. The CPV-455/2025 strain demonstrated 99.25% nucleotide identity with the QD-201 strain (Accession no. MT394166.1) identified in China in 2022 (Table 4).

### 2.3. Phylogenetic Evaluation of the Completed VP2 Gene Sequences of Vietnamese Parvovirus Strains

Phylogenetic analysis of the complete VP2 gene sequences, including the seven representative parvovirus strains and other sequences available in GenBank, revealed that the six parvovirus isolates were classified into three separate branches within the FPV group, while the CPV strain clustered within the CPV group, especially belonging to the canine CPV-2c lineages (Figure 1). More specifically, the FPV-343/2024 and FPV-351/2025 strains co-clustered within a distinct group and were closely related to the Beijing-I.3/2018 strain isolated from a cat in China in 2018 (Figure 1). Furthermore, these strains shared high genetic similarity with the previously reported Vietnamese FPV strains from 2018. Interestingly, four obtained FPV strains (FPV-340/2024, FPV-334/2024, -361/2025 and -465/2025) belonged to a separate branch of the FPV group. They showed close genetic relatedness to FPV strains reported in feline and canine hosts in China between 2018 and 2022 and Spain in 2023. Regarding CPV group, the CPV-455/2025 strain was positioned within a branch including Chinese and previously reported Vietnamese CPV strains from cats and dogs from the same period (Figure 1). The strain also shared high similarity with a Chinese strain reported in 2022 (Accession no. MT392166.1). In addition, several internal nodes had low-to-moderate bootstrap support; consequently, these relationships were interpreted as evidence of genetic relatedness rather than definitive evolutionary relationships.

### 2.4. Evaluation of Predicted aa Sequences of the Obtained Parvovirus VP2 Proteins

Analysis of the translated sequences indicated that seven FPV/CPV isolates were each 584 aa in length. Comparative analysis with the previously reported FPV Vietnamese from canines and cats, as well as vaccine strains, showed that the five-fold β-ribbon region (aa 152–169) was highly conserved across all analyzed FPV/CPV strains. However, multiple aa substitutions were observed in the N-terminal, Loop 1–Loop 4, and C-terminal regions (Table 5). In addition, residues at positions 101, 122, 236, 237, and 299 were T, N, T, D, and G, respectively. Notably, the FPV-343/2024, FPV-351/2025, CPV-455/2025, and FPV-465/2025 possessed A at position 91, whereas the FPV-334/2024, FPV-340/2024, and FPV-361/2025 retained S at this position. Furthermore, four of the current FPV/CPV strains exhibited V at position 232, while the CPV-455/2025 and FPV-465/2025 contained I at the same position.

### 2.5. Evaluation of Selective Pressure Patterns Across VP2 Protein Sequences of the Obtained FPV Strains

Selection pressure analysis identified evidence of negative selection at 26 sites within the VP2 protein of the analyzed FPV isolates, each with posterior probability values above 0.9 (Prob[α < β] > 0.9). In contrast, no sites under positive selection were detected in the VP2 protein (Table 6).

## 3. Discussion

Parvovirus, especially FPV, is one of the most transmissible and often fatal diseases affecting cats worldwide. Although vaccination is routinely practiced, parvovirus continues to be detected in domestic cats in Vietnam. Furthermore, genomic analyses have uncovered unique antigenic variations that distinguish FPV field strains from vaccine-derived strains [19].

In the present investigation, the genome of feline parvovirus was detected in 9.29% of fecal samples from domestic cats in two provinces/cities in Central Vietnam during 2023–2025. Interestingly, the study encompassed districts that had not previously been surveyed for feline parvovirus, thereby generating novel regional data. These findings help close epidemiological gaps and improve the overall surveillance infrastructure. Furthermore, the results showed that cats younger than 6 months exhibited a significantly greater rate of *Parvovirus* genome detection than cats aged 6 to 12 months (*p* < 0.05). This observation is in accordance with a previous research that three- to five-month-old kittens are particularly vulnerable to FPV [20]. However, the association between age and FPV/CPV positivity was based on univariable analysis and was not adjusted for potential confounding factors, particularly province in the current study. Therefore, the higher positivity observed in cats <6 months should be interpreted cautiously and does not necessarily indicate an independent effect of age. Future studies with larger sample sizes should use multivariable analyses to identify independent predictors of FPV infection. In addition, male and female positive rates were 7.96% and 10.26%, respectively, without a statistically validated difference (*p* > 0.05). This result indicates that sex does not influence the detection rate of feline parvovirus [21]. The present results are concordant with other studies reporting no meaningful differences in disease frequency based on sex or breed [21,22,23,24]. It is noted that vaccination history was unavailable for the sample cats in the present study. Therefore, the finding of viral DNA could not definitively distinguish natural FPV/CPV infection from potential vaccine-strain shedding or vaccine failure. This limitation should be considered when interpreting the prevalence and molecular findings of the present study.

Previous studies noted that a high rate of FPV-positive samples was detected in feces from domestic cats with diarrheal clinical signs in Iraq (38%), Iran (34%), and Bangladesh (22%). However, differences in diagnostic and epidemiological factors (sample types, study populations, clinical inclusion criteria, vaccination status, etc.) can substantially affect the probability of detecting FPV. They may partly explain the variation in reported detection rates. In the present study, the viral genome was detected in both clinical and non-clinical samples. Meanwhile Luan et al. (2026) [25] reported that out of 42 positive samples, only one was collected from a clinically healthy cat. In contrast, FPV shedding was observed in cats with or without clinical signs of feline panleukopenia. The authors found that among the positive samples, 60.3% (44/73) and 39.7% (29/73) were from clinically healthy and sick cats, respectively. Regarding subclinical field virus infections, viral shedding has been previously reported at various rates ranging from 4% to 37%. In the present study, we detected FPV DNA in both healthy (8.02%) and sick (14.04%) cats in Central Vietnam. Although FPV was detected more frequently in sick cats, the detection of viral DNA in clinically healthy cats indicated that the viral genome may be present without apparent clinical signs. It is noted that the current study is a cross-sectional study based on molecular detection; the presence of viral DNA in clinically healthy cats may not be interpreted as definitive evidence of active or prolonged subclinical shedding. It should be noted that other patterns, such as age, infection stage, viral load, vaccination history and sampling conditions, may contribute to an evaluated prevalence of subclinical shedding. Regarding the PCR assay, an extraction negative control was not included in the current study, limiting the assessment of contamination during DNA extraction. Nevertheless, this finding highlights the potential epidemiological importance of apparently healthy FPV/CPV-positive cats and underscores the need to implement stringent biosecurity measures to prevent infection associated with subclinical viral shedding in Central Vietnam.

The VP2-based phylogenetic tree indicated that six viruses were classified as FPV, whereas one isolate was assigned to the CPV lineage. This result reinforces the hypothesis that both FPV and CPV possess the capacity to infect feline populations and induce clinically similar disease manifestations [26]. Additionally, the observation is consistent with previous studies documenting cross-species transmission of CPV between canine and feline hosts [27,28,29]. Interestingly, other studies have proposed that the frequent close contact between cats and dogs may facilitate a cross-transmission cycle of FPV and CPV [30,31]. Notably, the identified FPV and CPV isolates clustered closely with Chinese reference strains. However, although the complete VP2 gene sequences shared close evolutionary relationships with Chinese isolates, the limited sample size and absence of epidemiological linkage data preclude definitive conclusions regarding potential cross-border transmission of FPV/CPV into Vietnam. The observed genetic similarity may reflect shared ancestry, widespread circulation of related viruses, sampling bias, limited phylogenetic resolution, or animal movement. These results point to the importance of strengthened regional surveillance strategies [32]. With respect to commercially available FPV vaccines, they rely on multidose schedules to achieve optimal immunity, which may increase the risk of infection in kittens during the period of incomplete protection. It is noteworthy that the continued global spread and evolution of FPV have resulted in substantial variability in genetic characteristics and virulence among circulating strains [33,34]. In this study, the Cat/Vietnam/FPV-465/2025 isolate clustered phylogenetically with the vaccine-related FPV strains, whereas the other detected FPV strains formed a distinct lineage separate from the vaccine group. It is noted that although the Cat/Vietnam/FPV-465/2025 strain was closely related to vaccine strains, the phylogenetic analysis could not determine whether the strain was of vaccine origin. It also should not be interpreted as evidence of vaccine failure or reduced vaccine efficacy. A previous study noted that FPV field strains circulating in China exhibited a high level of nucleotide identity to the 2008 Felocell vaccine strain, with full-length VP2 gene sequence identities ranging from 98.7% to 100%. This genetic similarity could be due to the frequent importation of dogs and cats from multiple countries, including China and parts of Europe (such as France and Italy), which facilitates the global dissemination of closely related strains. Another possibility is that this specific viral lineage has evolved and is widely circulating across Asian countries, including China and Central Vietnam [25]. It has been previously demonstrated that the vaccine effectiveness may be compromised for variant strains, as infections have been identified in vaccinated cats, reflecting poor cross-immunity against evolving viral variants [1]. Future studies should prioritize the isolation and comprehensive characterization of circulating field FPV strains, with a particular emphasis on elucidating the influence of viral genetic variability on the immunogenicity and protective efficacy of currently licensed commercial vaccines.

Recent evidence indicates that the VP2 gene of FPV is subject to progressive amino acid changes. The observed substitutions have influenced viral pathogenicity [35] and reduced vaccine effectiveness by modifying antigenic epitopes [36], thereby drawing significant attention. In agreement with these findings, the current study detected several amino acid changes in the VP2 protein. Notably, the I101T amino acid change in the VP2 protein is reported to influence structural configuration at the receptor-binding region by mediating polar interactions with Asp at position 99 [16]. This substitution may also reduce the effectiveness of neutralizing antibodies by modifying the spatial configuration of epitope. In the current study, all obtained strains exhibited the amino acid T at position 101. Structurally, the VP2 protein consists of four defined loops (1–4) along with a flexible loop [37]. Loops 1, 2, and 4 form the apical region of the threefold spike, whereas Loop 3 contributes to its shoulder. The flexible loop is essential for defining host range and hemagglutination capacity of FPV [38]. In addition, the A91S substitution observed in the study warrants particular attention. Predictive modeling indicates that this mutation could expand the random coil domain of the VP2 of FPV to encompass residues 91–95, leading to a structural transition of Loop 1 (aa 89–91) from an α-helix configuration to a flexible coil. This observed change may decrease neutralizing antibody interactions [39,40]. In contrast, the Loop 4 region (including site 440) exhibited a high level of conservation across all analyzed strains in the current study. Previous studies have demonstrated that the Loop 4 region of the VP2 protein of FPV/CPV is exposed on the virion surface, where it is implicated in host cell entry and serves as a major antigenic site targeted by neutralizing antibodies [41,42]. Substitutions at A91S, I101T, and residue 440 within the VP2 capsid protein of FPV/CPV may potentially influence electrostatic surface potential and epitope structure, thereby impairing antibody binding. These previous findings suggested that such mutations could be associated with immune evasion and host adaptation. In the present study, FPV exhibited a specific amino acid motif characterized by 232V-297V-300A-305A, which stands in clear contrast to the variant motif (232I-297A-300G-305Y) observed in CPV-2. This divergence at the molecular level is highly consistent with several previous investigations, which have collectively demonstrated that the amino acid residues at positions 80, 93, 103, 232, 323, 564, and 568 serve critical functional or structural roles. Ultimately, variations at these specific coordinates are instrumental in successfully differentiating FPV from CPV-2, and our data further validate their utility as reliable diagnostic or evolutionary markers. Additional studies focusing on both molecular mechanisms and immunological consequences are required to determine how these substitutions influence the efficacy of current vaccines.

Regarding the analysis of the natural selection profile, a previous study noted that the canine parvovirus VP2 protein had 50 and 41 sites of negative selection among CPV-2c and CPV-2b strains, respectively. The authors concluded that the complete VP2 protein of these CPV strains is under negative selection [41]. Similarly, Luan et al. (2026) noted that no positive selection sites were detected within the VP2 protein of feline parvovirus strains in China [25]. These authors also detected three peptides exhibiting strong epitope signals at positions 51–59, 242–250, and 284–292. Separately, another research group demonstrated that amino acid intervals 5–33 and 59–75 encompass linear B-cell epitopes. Furthermore, four distinct surface loops—Loop 1 (residues 84–94), Loop 2 (residues 216–235), Loop 3 (residues 295–306), and Loop 4 (residues 409–444)—contain the immunodominant domains previously mapped within the VP2 protein [43]. In the present study, we identified a total of 26 negative (purifying) selection sites among Central Vietnamese FPV strains. Notably, sites 57, 69, 221, 291, and 305 map directly to Loops 2 and 3, known B-cell epitopes, or strong-signal epitope regions. This evidence reveals that the VP2 gene and its encoded protein may potentially be undergoing strict purifying selection among these regional FPV strains. This evolutionary constraint limits adaptive diversification relative to more plastic viral evolutionary patterns, aligning consistently with previously described parvovirus dynamics [44]. However, the FUBAR analysis was based on a limited number of sequences, which may reduce its statistical power and robustness; therefore these findings should be considered preliminary and interpreted with caution. The present analysis also cannot determine whether these sites directly influence report binding, host range, antigenicity, or viral fitness. Further studies will be needed to evaluate the precise biological significance of these conserved sites.

This study has several limitations, including a relatively small sample size and restricted geographical sampling coverage. In addition, the virus was not isolated; therefore, its biological properties, antigenic characteristics, and pathogenic potential were not investigated, nor was the protective efficacy of currently available commercial vaccines assessed. Future investigations should incorporate larger sample sizes and broader geographic sampling to facilitate comprehensive analyses of the biological, antigenic, and pathogenic characteristics, as well as the complete genome sequences, of feline parvovirus. This will provide a more complete understanding of the epidemiological and molecular features of this virus across Vietnam.

## 4. Materials and Methods

### 4.1. Ethics Statement

The study protocol (CARE-2023/08) was approved on 25 March 2023, in accordance with ethical regulations of the Vietnam National University of Agriculture for animal handling and sample collection procedures. Approval was granted by the University’s Committee on Animal Research and Ethics. All sampling procedures were conducted to minimize animal stress and discomfort, consistent with internationally recognized animal welfare standards.

### 4.2. Sample Collection

A total of 269 fecal samples were obtained from domestic cats in Daklak and Gialai provinces in Central Vietnam over the period from July 2023 to July 2025. The cats were classified according to the presence or absence of vomiting and diarrhea. Cats showing either vomitting or diarrhea wer classified as having gastrointestinal clinical signs, whereas cats without reported vomitting or diarrhea were classified as having no reported gastrointestinal clinical signs. Samples were collected from different households, and no samples were repeatedly collected from the same household throughout the sampling period. However, all cats in the study had no vaccination information. The samples were homogenized in a 10% phosphate-buffered saline (*w*/*v*) and stored at −80 °C until further analysis.

### 4.3. DNA Extraction and PCR for the Feline Parvovirus Genome Detection

The Viral Gene-spin™ Viral DNA/RNA Extraction Kit (iNtRON Biotechnology, Seoul, Republic of Korea) was utilized to isolated total DNA from homogenates, following the procedures recommended by the manufacturer.

To detect *Parvovirus* DNA, Parvo-F/R primers were applied to amplify a 225 bp segment of VP2 gene (Table 7), as described previously. PCR amplification involved an initial denaturation at 95 °C for 5 min, followed by 35 cycles at 95 °C for 30 s, 60 °C for 60 s for detection, and extension at 72 °C for 60 s, with a final extension step at 72 °C for 10 min. Amplicons were resolved on 1.5% agarose gels and visualized using UV illumination. The positive samples were DNA extracted from a FELOCELL vaccine (Zoetis Inc., Lincoln, NE, USA), whereas the negative controls were distilled water.

### 4.4. Nucleotide Sequencing, Genetic and Phylogenetic Analysis

Sequencing was conducted using three primer pairs to obtain PCR products of 708 bp, 719 bp, and 569 bp (Table 7). The primers target conserved regions of the VP2 genes of FPV and homologous VP2 regions of closely related CPV strains. PCR amplification involved an initial denaturation at 95 °C for 5 min, followed by 40 cycles at 95 °C for 30 s, 52 °C for 30 s, and extension at 72 °C for 60 s, with a final extension step at 72 °C for 10 min. Amplicons were resolved on 1.5% agarose gels and visualized using UV illumination.

The sequencing of the PCR products was carried out by 1st BASE (Selango, Malaysia). Each amplicon segment was sequenced in duplicate. The sequences obtained were aligned and compared with those available in GenBank (Table 8) by using GENETYX version 10 software (GENETYX Corp., Tokyo, Japan) and BLAST analysis (https://blast.ncbi.nlm.nih.gov/Blast.cgi). Deduced amino acid sequences were analyzed using the Clustal W algorithm within the BioEdit version 7.2 [46,47]. Phylogenetic analysis was performed using the maximum likelihood method with 1000 bootstrap replicates in MEGA 7 [48]. The Tamura-3 parameter model incorporating a gamma distribution and invariant sites (T92 + G + I) was selected as the best-fit model.

### 4.5. Analysis of Selection Profiles

Datamonkey (http://www.datamonkey.org/) was applied to evaluate the evolutionary selection profiles among feline parvoviruses in this study. The analysis was determined using the Fast Unconstrained Bayesian AppRoximation (FUBAR) method [49].

### 4.6. Nucleotide Sequence Accessions Numbers

The full-length VP2 sequences of feline-derived FPV/CPV strains in this study were deposited into GenBank under the accession numbers PZ276925 to PZ276931.

### 4.7. Statistical Analysis

A two-sided Fisher’s exact test was applied to assess differences in feline parvovirus-positive genome detection rates between geographical regions and other criteria (breed, sex, and health status). For age, Pearson’s chi-square test followed by two-sided pairwise Fisher’s exact tests were used. For two-group comparison, odds ratios (Ors) with 95% confidence intervals (CIs) were calculated. A *p*-value < 0.05 was considered statistically significant.

## 5. Conclusions

Overall, this research provides the first molecular characterization of the FPV/CPV in domestic cats sampled in the Daklak and Gialai provinces in Central Vietnam in 2025 and presents a detailed molecular characterization of FPV/CPV strains circulating in this region. The study provides valuable data for advancing research on parvovirus evolution and underscores the critical role of genetic approaches in understanding viral diversity.

## Figures and Tables

**Figure 1:**
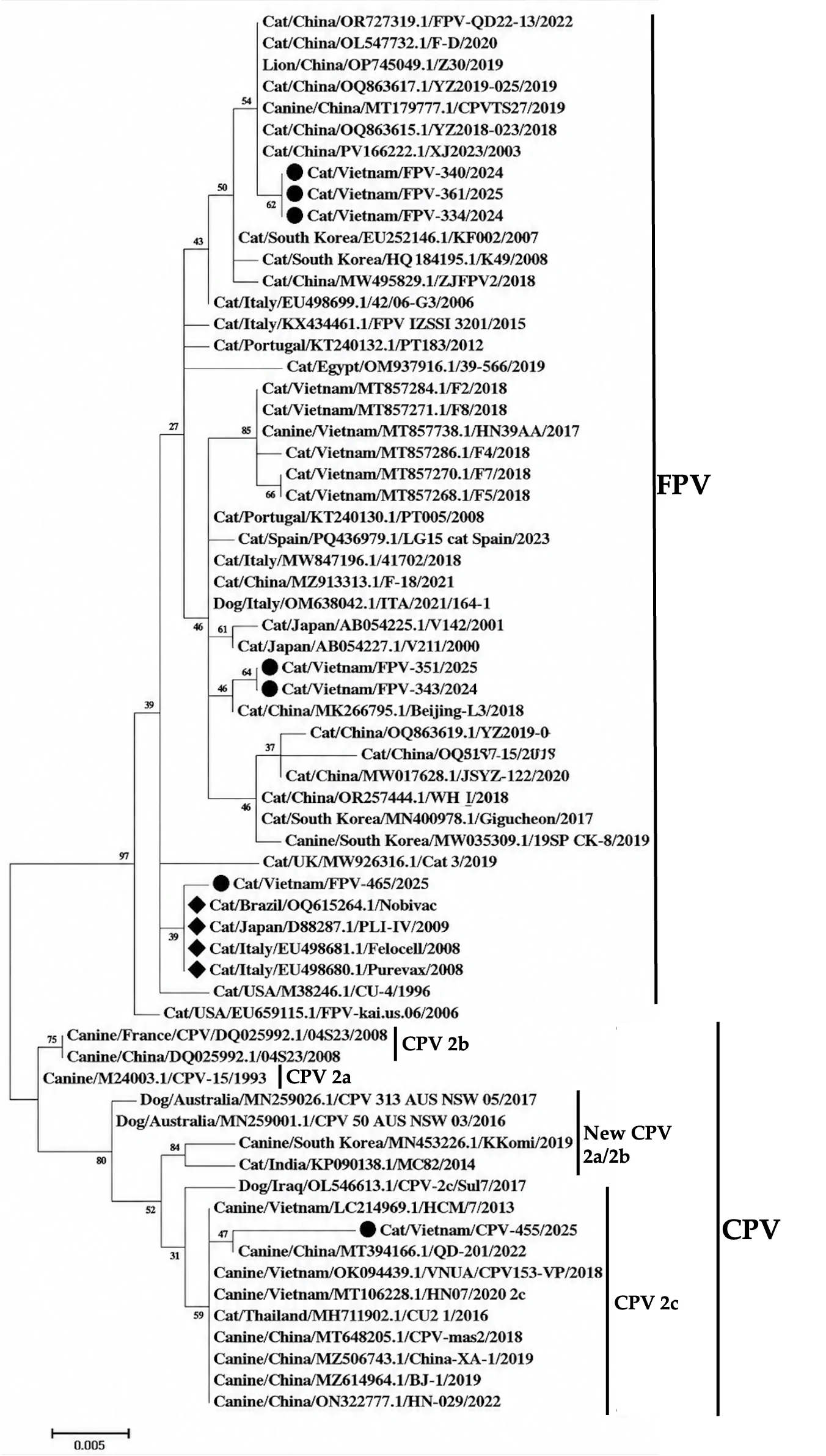
A phylogenetic tree was constructed based on the complete VP2 gene of Vietnamese FPV/CPV sequences (1755 bp) and reference strains obtained from GenBank. The phylogenetic tree was inferred using the maximum likelihood method implemented in MEGA7 (https://megasoftware.net/), applying the best-fit nucleotide substitution model of Tamura 3-parameter with gamma-distributed rate variation and invariant sites. Node support was evaluated with 1000 bootstrap replicates. Bootstrap values > 50% are displayed at the nodes. FPV/CPV strains identified in the current study are denoted by black circles, whereas the vaccine strains are marked with black diamonds.

**Table 1:** Identification of the *Parvovirus* genome in the obtained samples by using the conventional PCR method.

Province/City	No. of Examined Cases	No. of Positive Cases (%)	95% CI
Daklak	213	11 (5.16 ^a^)	2.51–8.73
Gialai	56	14 (25.00 ^b^)	14.39–38.37
Total	269	25 (9.29)	5.93–13.03

^a,b^: indicates a statistically meaningful difference in the *Parvovirus* infection (*p* < 0.05). The comparisons among province status were exploratory; no adjustment for multiple testing across the demographic variables was applied.

**Table 2:** An evaluation of parvovirus genome detection in the obtained samples by breed, age, sex, and health status.

Criteria		No. of Examined Cases	No. of Positive Cases	Positive Rate (%)	95% CI
Breed	Native cats	216	23	10.65	6.87–15.55
	Exotic, cross-breed cats	53	2	3.77	0.46–12.98
Age (Months)	<6	85	15	17.65 ^a^	10.23–27.43
	6–12	118	5	4.24 ^b^	1.39–9.61
	>12	66	5	7.58 ^ab^	2.51–16.80
Sex	Male	113	9	7.96	3.71–14.58
	Female	156	16	10.26	5.98–16.12
Health Status	Healthy	212	17	8.02	4.74–12.53
	Sick	57	8	14.04	6.26–25.79

Different superscript letters indicated significant differences between groups (*p* < 0.05), whereas groups sharing at least one superscript letter do not differ significantly. The comparisons among breed, age, sex, and health status were exploratory; no adjustment for multiple testing across these demographic variables was applied.

**Table 3:** (**A**). The comparative nucleotide identities of the Vietnamese parvovirus strains identified in this study. (**B**). The comparative predicted amino acid (aa) identities of the Vietnamese parvovirus strains identified in this study.

(**A**)								
**Strain Name**	**Province**	**FPV-334/2024**	**FPV-340/2024**	**FPV-343/2024**	**FPV-351/2025**	**FPV-361/2025**	**CPV-455/2025**	**FPV-465/2025**
FPV-334/2024	Gialai	100						
FPV-340/2024	Gialai	100	100					
FPV-343/2024	Gialai	99.37	99.37	100				
FPV-351/2025	Daklak	99.37	99.37	100	100			
FPV-361/2025	Daklak	100	100	99.37	99.37	100		
CPV-455/2025	Daklak	98.68	98.68	98.63	98.63	98.68	100	
FPV-465/2025	Daklak	98.74	98.74	98.68	98.68	98.74	98.23	100
(**B**)								
**Strain Name**	**Province**	**FPV-334/2024**	**FPV-340/2024**	**FPV-343/2024**	**FPV-351/2025**	**FPV-361/2025**	**CPV-455/2025**	**FPV-465/2025**
FPV-334/2024	Gialai	100	100	99.38	99.38	100	98.68	98.75
FPV-340/2024	Gialai		100	99.38	99.38	100	98.68	98.75
FPV-343/2024	Gialai			100	100	99.38	98.63	98.70
FPV-351/2025	Daklak				100	99.38	98.63	98.70
FPV-361/2025	Daklak					100	98.68	98.75
CPV-455/2025	Daklak						100	98.22
FPV-465/2025	Daklak							100

**Table 4:** The characteristics of the obtained Vietnamese parvovirus in the study.

Strain Name (GenBank Accession Number)	Virus with the Highest Nucleotide Identity (%)				
	GenBank Accession Number	Country	Strain	Year	Rate (%)
Cat/Vietnam/FPV-334/2024 (PZ276925)	OQ863617.1	China	YZ2019-025	2019	99.94
Cat/Vietnam/FPV-340/2024 (PZ276926)	OQ863617.1	China	YZ2019-025	2019	99.94
Cat/Vietnam/FPV-343/2024 (PZ276927)	MW017628.1	China	JSYZ-122	2020	99.77
Cat/Vietnam/FPV-351/2025 (PZ276928)	MW017628.1	China	JSYZ-122	2020	99.77
Cat/Vietnam/FPV-361/2025 (PZ276929)	OQ863617.1	China	YZ2019-025	2019	99.94
Cat/Vietnam/CPV-455/2025 (PZ276931)	MT394166.1	China	QD-201	2022	99.25
Cat/Vietnam/FPV-465/2025 (PZ276930)	EU498681.1	Italy	Felocell	2008	99.85

**Table 5:** An analysis of amino acid replacements in the VP2 protein of the parvovirus isolates (**A**,**B**).

(**A**)							
Strain name	Amino acid sites in the VP2 protein						
	N terminatal region (1–37)			Loop 1 (84–94)			Loop 2 (216–235)
	5			87	91	93	232
	A			M	S	N	V
Cat/Vietnam/FPV-334/2024	.			.	.	K	.
Cat/Vietnam/FPV-340/2024	.			.	.	K	.
Cat/Vietnam/FPV-343/2024	.			.	A	K	.
Cat/Vietnam/FPV-351/2025	.			.	A	K	.
Cat/Vietnam/FPV-361/2025	.			.	.	K	.
Cat/Vietnam/CPV-455/2025	.			.	A	K	I
Cat/Vietnam/FPV-465/2025	G			L	A	.	I
Previous Vietnamese Parvovirus in Cats ^a^	A/G			M/L	A	.	V/I
Vaccine Parvovirus Strains in Cats ^b^	.			.	A	.	I
(**B**)							
Strain name	Amino acid sites in the VP2 protein						
	Loop 3 (295–306)			Loop 4 (409–444)	C terminal region (500–584)		
	297	300	305	426	562	564	568
	S	A	D	N	V	N	A
Cat/Vietnam/FPV-334/2024	.	.	.	.	.	.	.
Cat/Vietnam/FPV-340/2024	.	.	.	.	.	.	.
Cat/Vietnam/FPV-343/2024	.	.	.	.	.	.	.
Cat/Vietnam/FPV-351/2025	.	.	.	.	.	.	.
Cat/Vietnam/FPV-361/2025	.	.	.	.	.	.	.
Cat/Vietnam/CPV-455/2025	A	G	Y	.	.	.	A
Cat/Vietnam/FPV-465/2025	.	.	.	E	.	.	.
Previous Vietnamese Parvovirus in Cats ^a^	S/A	A/G	D/Y	V/E	.	V/S	A/G
Vaccine Parvovirus Strains in Cats ^b^	.	.	.	.	L	.	.

^a^ Representative previous Vietnamese FPV/CPV strains, such as Canine/Vietnam/HCM/7/2013 (LC214969); Canine/Vietnam/HN39AA/2017 (MK357738); Cat/Vietnam/F5/2018 (MT857268); Cat/Vietnam/F4/2018 (MT857286); Cat/Vietnam/F2/2018 (MT857284); Canine/Vietnam/VNUA/CPV153-VP/2018 (OK094439); Canine/Vietnam/HN07/2020 2c (MT106228). ^b^ Cat/Brazil/Nobivac (OQ615264), Cat/Japan/PLI-IV/2009 (D88287.1), Cat/Italy/Felocell/2008 (EU498681), Cat/Italy/Purevax/2008 (EU498680). The dots “.” indicate values that are the same as the representative FPV sequences.

**Table 6:** The negative selection profile of the VP2 protein sequences of Vietnamese FPV strains.

No.	Site	α	β	α-β	Prob[α > β]	Prob[α < β]	Bayes Factor [α < β]
1	45	25.993	1.432	−24.561	0.934	0.048	0.075
2	57	31.034	1.164	−29.87	0.961	0.028	0.043
3	69	27.961	1.356	−26.605	0.944	0.041	0.063
4	82	27.961	1.356	−26.605	0.944	0.041	0.063
5	152	30.924	1.298	−29.625	0.957	0.031	0.047
6	159	24.251	1.635	−22.616	0.921	0.058	0.092
7	164	31.948	1.294	−30.654	0.961	0.028	0.042
8	171	28.008	1.284	−26.724	0.946	0.039	0.06
9	172	4.694	1.607	−3.087	0.948	0.401	0.989
10	193	30.079	1.296	−28.783	0.954	0.033	0.051
11	221	31.904	1.501	−30.403	0.957	0.03	0.045
12	250	23.212	1.299	−21.913	0.924	0.057	0.09
13	291	26.291	1.682	−24.609	0.929	0.051	0.08
14	305	31.984	1.277	−30.707	0.961	0.028	0.042
15	342	31.228	1.546	−29.683	0.953	0.033	0.05
16	347	28.008	1.284	−26.724	0.946	0.039	0.06
17	364	22.482	1.278	−21.204	0.921	0.059	0.094
18	383	30.085	1.641	−28.444	0.947	0.037	0.058
19	391	29.763	1.297	−28.466	0.953	0.034	0.052
20	395	30.978	1.337	−29.641	0.959	0.029	0.044
21	430	25.749	1.721	−24.028	0.926	0.054	0.084
22	492	31.776	1.289	−30.486	0.96	0.028	0.043
23	503	23.398	1.28	−22.118	0.926	0.055	0.087
24	522	30.407	1.301	−29.106	0.958	0.03	0.045
25	541	29.711	1.285	−28.426	0.953	0.034	0.052
26	553	29.518	1.297	−28.221	0.952	0.035	0.053

α: represents the posterior rate of synonymous substitution at a given site; β: defines the posterior rate of nonsynonymous substitutions. Selection regimes are inferred as follows: α > β indicates purifying selection, α < β indicates diversifying selection, and α = β suggests neutral selection. A posterior probability threshold of 0.9 is employed, where Prob[α > β] ≥ 0.9 supports negative selection and Prob[α < β] ≥ 0.9 supports positive selection at a given site.

**Table 7:** The primer sets used in the present study.

Name	Primer Sequence (5′–3′)	Fragment Size (bp)	References
Parvo-F	TGC CTC AAT CTG AAG GAG CT	225	[23]
Parvo-R	TTT CAT CTG TTT GCG CTC CC		
S1-F	CCA ACT AAA AGA AGT AAA CC	708	[13,17,45]
S1-R	TGG TTG GTT TCC ATG GAT AAA AAC C		
S2- F	AGA TAG TAA TAA TAC GCC ATT T	719	
S2-R	TTT TGA ATCC AAT CTC CTT CTG GAT		
S3-F	ACA GGA GAA ACA CCT GAG AGA TTT A	569	
S3-R	AGG TGC TAG TTG AGA TTT TTC AT		

**Table 8:** A list of the parvovirus-VP2 sequences used in this study.

No.	GenBank Accession No.	Strain Name	Country	Source	Year
1	MW495829.1	ZJFPV2	China	Cat	2018
2	OR257444.1	WH 1	China	Cat	2018
3	OR727319	QD22-13	China	Cat	2022
4	OL547732	F-D	China	Cat	2020
5	OP745049	Z30	China	Panthera leo	2019
6	MZ913313.1	F-18	China	Cat	2021
7	DQ025992.1	04S23	China	Canine	2008
8	MT648205.1	CPV-mas2	China	Canine	2018
9	MZ614964.1	BJ-1	China	Canine	2019
10	MZ506743.1	China-XA-1	China	Canine	2019
11	ON322777.1	HN-029	China	Canine	2022
12	MT394166.1	QD-201	China	Canine	2022
13	OQ863617	YZ2019-025	China	Cat	2019
14	MT857284.1	F2	Vietnam	Cat	2018
15	MT857286.1	F4	Vietnam	Cat	2018
16	MT857268.1	F5	Vietnam	Cat	2018
17	MT857270.1	F7	Vietnam	Cat	2018
18	MT857271.1	F8	Vietnam	Cat	2018
19	LC214969.1	HCM/7	Vietnam	Canine	2013
20	OK094439.1	VNUA/CPV153-VP	Vietnam	Canine	2018
21	MT106228.1	HN07	Vietnam	Canine	2020
22	EU498699.1	42/06-G3	Italy	Cat	2006
23	EU498680.1	Purevax	Italy	Cat	2008
24	EU498681.1	Felocell	Italy	Cat	2008
25	KX434461.1	FPV_IZSSI_3201	Italy	Cat	2015
26	MW847196.1	41702	Italy	Cat	2018
27	OM638042.1	164-1	Italy	Canine	2021
28	AB054227.1	V211	Japan	Cat	2000
29	AB054225.1	V142	Japan	Cat	2001
30	D88287.1	PL-IV	Japan	Cat	2009
31	EU252146.1	KF002	Republic of Korea	Cat	2007
32	HQ184195.1	K49	Republic of Korea	Cat	2008
33	MN400978.1	Gigucheon	Republic of Korea	Cat	2017
34	MW035309.1	19SP_CK-8	Republic of Korea	Canine	2019
35	MN453226.1	KKomi	Republic of Korea	Canine	2019
36	OQ615264.1	Nobivac	Brazil	Cat	2008
37	MT179777	CPVTS27	China	Cat	2019
38	KT240132.1	PT183	Portugal	Cat	2012
39	MH711902.1	CU21	Thailand	Cat	2016
40	MW926316.1	Cat 3	UK	Cat	2019
41	PQ436979.1	LG15_cat_Spain	Spain	Cat	2023
42	OQ863615	YZ2018-023	China	Cat	2018
43	OM937916.1	FPV-AUS-14	Egypt	Cat	2017
44	M38246.1	CU-4	USA	Cat	1996
45	EU659115.1	FPV-kai.us.06	USA	Cat	2006
46	M24003.1	CPV-15	USA	Canine	1993
47	KP090138.1	MC8	India	Cat	2014
48	PV166222	XJ2023	China	Cat	2023
49	MT857738.1	HN39AA	Vietnam	Cat	2017
50	KT240130	PT005/08	Portugal	Cat	2008
51	MK266795	Beijing-L3/2018	China	Cat	2017
52	MW017628	JSYZ-122	China	Cat	2020
53	OQ863619	YZ2019-027	China	Cat	2019
54	OQ815872	FPV-15	China	Cat	2018
55	MN259026	CPV_313_AUS_NSW_05/2017	Australia	Cat	2017
56	MN259001	CPV_50_AUS_NSW_03/2016	Australia	Cat	2016
57	OL546613	CPV-2c/SuL7/2017	Iraq	Cat	2017

## Data Availability

The original contributions presented in this study are included in the article. Further inquiries can be directed to the corresponding author.

## References

[1] Barrs V.R. (2019). Feline Panleukopenia: A Re-emergent Disease. Vet. Clin. N. Am. Small Anim. Pract..

[2] Kruse B.D., Unterer S., Horlacher K., Sauter-Louis C., Hartmann K. (2010). Prognostic Factors in Cats with Feline Panleukopenia. J. Vet. Intern. Med..

[3] Cave T.A., Thompson H., Reid S.W.J., Hodgson D.R., Addie D.D. (2002). Kitten mortality in the United Kingdom: A retrospective analysis of 274 histopathological examinations (1986 to 2000). Vet. Rec..

[4] Raheena K.P., Priya P.M., Mani B.K., Mini M., Pillai U.N. (2016). Comparison of different diagnostic test to detect feline panleukopenia virus among cats in Kerala, India. Indian J. Anim. Res..

[5] Sykes J.E. (2014). Feline Panleukopenia Virus Infection and Other Viral Enteritides. Canine Feline Infect. Dis..

[6] Tuzio H., Miller L., Janeczko S., Hurley K.F. (2021). Feline Panleukopenia. Infectious Disease Management in Animal Shelters.

[7] Truyen U., Addie D., Belák S., Boucraut-Baralon C., Egberink H., Frymus T., Gruffydd-Jones T., Hartmann K., Hosie M.J., Lloret A. (2009). Feline panleukopenia. ABCD guidelines on prevention and management. J. Feline Med. Surg..

[8] Horiuchi M., Yamaguchi Y., Gojobori T., Mochizuki M., Nagasawa H., Toyoda Y., Ishiguro N., Shinagawa M. (1998). Differences in the evolutionary pattern of feline panleukopenia virus and canine parvovirus. Virology.

[9] Carmichael L.E., Joubert J.C., Pollock R.V. (1980). Hemagglutination by canine parvovirus: Serologic studies and diagnostic applications. Am. J. Vet. Res..

[10] Johnson R.H., Spradbrow P.B. (1979). Isolation from dogs with severe enteritis off a parvovirus related to Feline Panleucopaenia virus. Aust. Vet. J..

[11] Siegl G., Bates R.C., Berns K.I., Carter B.J., Kelly D.C., Kurstak E., Tattersall P. (1985). Characteristics and taxonomy of Parvoviridae. Intervirology.

[12] Balboni A., Bassi F., De Arcangeli S., Zobba R., Dedola C., Alberti A., Battilani M. (2018). Molecular analysis of carnivore Protoparvovirus detected in white blood cells of naturally infected cats. BMC Vet. Res..

[13] Yang D.-K., Park Y.-R., Park Y., An S., Choi S.-S., Park J., Hyun B.-H. (2022). Isolation and Molecular Characterization of Feline panleukopenia Viruses From Korean Cats. Korean J. Vet. Res..

[14] Chang D., Liu Y., Chen Y., Hu X., Burov A., Puzyr A., Bondar V., Yao L. (2020). Study of the immunogenicity of the VP2 protein of canine parvovirus produced using an improved Baculovirus expression system. BMC Vet. Res..

[15] Su W.J., Shen W.D., Li B., Wu Y., Gao G., Wang W.B. (2009). A novel way to purify recombinant baculoviruses by using bacmid. Biosci. Rep..

[16] Hoang M., Wu C.N., Lin C.F., Nguyen H.T.T., Le V.P., Chiou M.T., Lin C.N. (2020). Genetic characterization of feline panleukopenia virus from dogs in Vietnam reveals a unique Thr101 mutation in VP2. PeerJ.

[17] Dang T.T.M., Tran T.T., Van T.M., Le Q.T., Tran N.B. (2023). First molecular report of feline panleukopenia virus infection in diarrheic cats at Can Tho City, Vietnam. Vet. Integr. Sci..

[18] Nguyen Y.T., Nguyen S.V., Nguyễn N.T., Le H.V., Nguyen G.T., Pham H.Q. (2021). Pathological features and biological characteristics of feline panleukopenia virus isolate from cat in Hanoi. Viet. J. Agric. Sci..

[19] Raja P., Mallika K.S., Viva V.Y., Parthiban M., Sathish G., Vinitha V., Parthiban S., Raj G.D. (2024). Complete genome sequence and phylogenetic analysis of feline panleukopenia virus from India. Virusdisease.

[20] Kruse B.D., Unterer S., Horlacher K., Sauter-Louis C., Hartmann K. (2011). Feline panleukopenia-different course of disease in cats younger than versus older than 6 months of age?. Tierarztl. Prax. Ausg. K Kleintiere Heimtiere.

[21] Ullah M.N., Gofur M.R., Sarder M.J.U. (2025). Antigenic characterization and clinical manifestations of feline Panleukopenia virus in Cats. Vet. Res. Notes.

[22] Jeba N., Mia R., Tarafder M.M.B., Mozumder A., Farazi R., Hasan S.M.N., Bostami M.B., Rahman A., Mannan A., Akter S. (2025). Comparative Detection and Genetic Characterization of Feline Panleukopenia Virus in Bangladesh. Vet. Med. Sci..

[23] Awad R.A., Khalil W.K.B., Attallah A.G. (2018). Epidemiology and diagnosis of feline panleukopenia virus in Egypt: Clinical and molecular diagnosis in cats. Vet. World.

[24] Islam M.A., Rahman M.S., Rony S.A., Uddin M.J., Rahman A.K.M.A. (2010). Antigenic detection of feline panleukopenia virus in local breed cats at tangail district in Bangladesh. Int. J. BioRes..

[25] Luan Q., Weng C., Guo Y., Yang P., Wu G., Ding G., Li Y. (2026). Genetic variability and phylogenetic analysis of the VP2 gene of feline parvovirus in Harbin. Front. Vet. Sci..

[26] Battilani M., Balboni A., Ustulin M., Giunti M., Scagliarini A., Prosperi S. (2011). Genetic complexity and multiple infections with more Parvovirus species in naturally infected cats. Vet. Res..

[27] Miranda C., Parrish C.R., Thompson G. (2014). Canine parvovirus 2c infection in a cat with severe clinical disease. J. Vet. Diagn. Investig..

[28] Li X., Wu H., Wang L., Spibey N., Liu C., Ding H., Liu W., Liu Y., Tian K. (2018). Genetic characterization of parvoviruses in domestic cats in Henan province, China. Transbound. Emerg. Dis..

[29] Jing Z., Ji P., Wei Y., Hao F., Wei Y. (2022). Isolation and identification of a novel canine parvovirus type 2c strain in domestic cats in Dalian, China. Front. Vet. Sci..

[30] Truyen U., Evermann J.F., Vieler E., Parrish C.R. (1996). Evolution of canine parvovirus involved loss and gain of feline host range. Virology.

[31] Chen S., Shang K., Chen J., Yu Z., Wei Y., He L., Ding K. (2024). Global distribution, cross-species transmission, and receptor binding of canine parvovirus-2: Risks and implications for humans. Sci. Total Environ..

[32] Chowdhury Q., Alam S., Chowdhury M.S.R., Hasan M., Uddin M.B., Hossain M.M., Islam M.R., Rahman M.M., Rahman M.M. (2021). First molecular characterization and phylogenetic analysis of the VP2 gene of feline panleukopenia virus in Bangladesh. Arch. Virol..

[33] Zhang H., Zhang W., Pan Y., Li H., He T., Dong Q., Song W., Zhang W., Zhang L., Kareem K. (2024). Evolutionary Dynamics and Pathogenicity Analysis of Feline Panleukopenia Virus in Xinjiang, China. Microorganisms.

[34] Chung H.C., Kim S.J., Nguyen V.G., Shin S., Kim J.Y., Lim S.K., Park Y.H., Park B. (2020). New genotype classification and molecular characterization of canine and feline parvoviruses. J. Vet. Sci..

[35] Yu Z., Wang W., Yu C., He L., Ding K., Shang K., Chen S. (2024). Molecular Characterization of Feline Parvovirus from Domestic Cats in Henan Province, China from 2020 to 2022. Vet. Sci..

[36] Wen Y., Tang Z., Wang K., Geng Z., Yang S., Guo J., Chen Y., Wang J., Fan Z., Chen P. (2024). Epidemiological and Molecular Investigation of Feline Panleukopenia Virus Infection in China. Viruses.

[37] Parker J.S., Parrish C.R. (1997). Canine parvovirus host range is determined by the specific conformation of an additional region of the capsid. J. Virol..

[38] Simpson A.A., Chandrasekar V., Hébert B., Sullivan G.M., Rossmann M.G., Parrish C.R. (2000). Host range and variability of calcium binding by surface loops in the capsids of canine and feline parvoviruses. J. Mol. Biol..

[39] Wang J., Yan Z., Liu H., Wang W., Liu Y., Zhu X., Tian L., Zhao J., Peng Q., Bi Z. (2024). Prevalence and Molecular Evolution of Parvovirus in Cats in Eastern Shandong, China, between 2021 and 2022. Transbound. Emerg. Dis..

[40] Chen X., Wang J., Zhou Y., Yue H., Zhou N., Tang C. (2022). Circulation of heterogeneous Carnivore protoparvovirus 1 in diarrheal cats and prevalence of an A91S feline panleukopenia virus variant in China. Transbound. Emerg. Dis..

[41] Alexis V.A., Sonia V., Daniela S., Miguel G., Timothy H., Valentina F., Lisette L., Leonardo S. (2021). Molecular Analysis of Full-Length VP2 of Canine Parvovirus Reveals Antigenic Drift in CPV-2b and CPV-2c Variants in Central Chile. Animals.

[42] Wu Q., Jin Y., Cao W., Ren Z., Li X., Li Z., Han J., Shi C., Gao R., Yan M. (2025). Engineering a recombinant VP2-Based neutralizing epitope vaccine candidate against canine parvovirus: A preliminary immunogenicity assessment. Vet. Res. Commun..

[43] Safwat M.S., Xi C., M S.E., Anwer A.Z., Ali M.E., Abdallah E.S.A., Amer H.M., Saeed O.S., Khalil G.M., Abdelhaleem S.W. (2025). Emergence, surge, and fading of the novel feline parvovirus Thr390Ala mutant in Egyptian cats during 2023: Insights from a comprehensive full-length VP2 genetic analysis. BMC Vet. Res..

[44] Parrish C.R. (1999). Host range relationships and the evolution of canine parvovirus. Vet. Microbiol..

[45] Kaur G., Chandra M., Dwivedi P.N., Sharma N.S. (2015). Isolation of Canine parvovirus with a view to identify the prevalent serotype on the basis of partial sequence analysis. Vet. World.

[46] Hall T.A. (1999). BioEdit: A user-friendly biological sequence alignment editor and analysis program for Windows 95/98/NT. Nucleic Acids Symp. Ser..

[47] Thompson J.D., Higgins D.G., Gibson T.J. (1994). CLUSTAL W: Improving the sensitivity of progressive multiple sequence alignment through sequence weighting, position-specific gap penalties and weight matrix choice. Nucleic Acids Res..

[48] Kumar S., Stecher G., Tamura K. (2016). MEGA7: Molecular Evolutionary Genetics Analysis Version 7.0 for Bigger Datasets. Mol. Biol. Evol..

[49] Murrell B., Moola S., Mabona A., Weighill T., Sheward D., Kosakovsky Pond S.L., Scheffler K. (2013). FUBAR: A fast, unconstrained bayesian approximation for inferring selection. Mol. Biol. Evol..

